# Chromatin accessibility profile and the role of PeAtf1 transcription factor in the postharvest pathogen *Penicillium expansum*

**DOI:** 10.1093/hr/uhae264

**Published:** 2024-09-20

**Authors:** Yiran Wang, Kaili Wang, Qiya Yang, Zhaoting Wang, Yingying Su, Xifei Chen, Hongyin Zhang

**Affiliations:** School of Food and Biological Engineering, Jiangsu University, Zhenjiang 212013, Jiangsu, China; School of Food and Biological Engineering, Jiangsu University, Zhenjiang 212013, Jiangsu, China; School of Food and Biological Engineering, Jiangsu University, Zhenjiang 212013, Jiangsu, China; School of Food and Biological Engineering, Jiangsu University, Zhenjiang 212013, Jiangsu, China; School of Food and Biological Engineering, Jiangsu University, Zhenjiang 212013, Jiangsu, China; School of Food and Biological Engineering, Jiangsu University, Zhenjiang 212013, Jiangsu, China; School of Food and Biological Engineering, Jiangsu University, Zhenjiang 212013, Jiangsu, China

## Abstract

Gene transcription is governed by a complex regulatory system involving changes in chromatin structure, the action of transcription factors, and the activation of *cis*-regulatory elements. Postharvest fruits are threatened by *Penicillium expansum*, a leading causal agent of blue mold disease and one of the most economically significant postharvest pathogens worldwide. However, information on its transcription regulatory mechanism is lagging. Here, we conducted an assay for transposase accessible chromatin sequencing (ATAC-seq) for *P. expansum* during vegetative growth and infection phase and then studied the function of a basic leucine zipper (bZIP) transcription factor PeAtf1. Results highlighted the role of promoter regions in gene transcription and the significant difference in *P. expansum* between these two phases. Six footprint-supported *cis*-regulatory elements of active transcription factors were obtained and analyzed. We then identified a homolog of the bZIP regulator Atf1, PeAtf1, and found it positively regulated vegetative growth, reproduction, and osmotic stress response in *P. expansum*. Furthermore, *PeAtf1* deletion enhanced the fungus's tolerance to oxidative, cell wall, and membrane stresses, which might contribute to the virulence of deletion mutants in apple fruits, leading to similar pathogenicity between mutants and the wild type. Overall, this study provides new insights into the transcription regulatory profile of *P. expansum*, aiding in the future development of strategies to control *P. expansum*.

## Introduction

The dynamic changes in chromatin structure play a crucial role in the regulation of transcription in eukaryotic organisms. Accessible chromatin (or open chromatin) at promoters, enhancers, and other transcription regulatory regions facilitates the binding of transcription factors to *cis*-regulatory elements, thereby controlling gene transcription [1]. Identifying these regions can enhance our understanding of the transcriptional regulatory network and the information on *cis*-regulatory elements to which transcription factors bind [2]. The assay for transposase accessible chromatin sequencing (ATAC-seq) is a method used to analyze chromatin accessibility on a genome-wide scale by integrating Tn5 transposase into open chromatin regions [3]. It can not only capture regions of open chromatin but also infer potential binding sites of active transcription factors through footprint-supported motifs [4]. Compared to other chromatin sequencing technologies, such as deoxyribonuclease I hypersensitivity sequencing (DNase-seq) and micrococcal nuclease digestion with sequencing (MNase-seq), ATAC-seq demonstrates advantages in terms of sample requirements, repeatability, and efficiency [5]. Therefore, in recent years, this approach has been widely utilized to identify open chromatin and motif characteristics in both animals and plants [6–8]. However, its application in fungi, especially plant pathogens, is limited due to the issue of contamination and the difficulty of isolating fungal nuclei from plant tissues [4, 9, 10].

*Penicillium expansum* is a prevalent and devastating pathogen in the postharvest field. In addition to its primary hosts, apples and pears, it can infect >20 fruits and vegetables, including strawberries, kiwifruits, carrots, and tomatoes [11–13]. Not only is it the primary pathogen behind fruit spoilage, but it is also a major producer of the mycotoxin patulin in the food supply, resulting in food safety concerns [14]. Since *P. expansum* is a severe threat to the fruit and fruit-derived industries, understanding the molecular mechanisms by which it grows and infects hosts is the basis for creating effective management strategies. So far, in *P. expansum*, advancements in next-generation sequencing have allowed researchers to explore the biological characteristics and genomic features of this fungus [12, 15]. Our previous study identified numerous genes with differential expression in *P. expansum* during vegetative growth and infection phase at the transcriptional level [16]. The expression level of these genes is controlled by a complex regulatory system influenced by chromatin structure and transcription factors. But our understanding of the transcriptional regulatory mechanisms in *P. expansum* is lagging.

Whole-genome sequencing initially revealed distinct transcription factor characteristics in *P. expansum*, including the C_2_H_2_ type, basic leucine zipper (bZIP), basic helix–loop–helix (bHLH), and Zn(II)_2_Cys_6_ (C_6_) families [15]. PeSte12, a C_2_H_2_-type zinc finger protein involved in the mitogen-activated protein kinase (MAPK) signaling pathway, has been proven to act as a positive regulator of sporulation and pathogenicity in *P. expansum* [17]. Subsequently, another member of the C_2_H_2_ family, PeBrlA, was identified and found to participate in conidia formation and penicillin production in *P. expansum* [18]. The bZIP family is among the largest, most widely distributed, and structurally conserved transcription factor families in nearly all eukaryotes. Nineteen members from the bZIP family exist in the *P. expansum* genome [15]. The bZIP proteins are identified by a conserved DNA-binding region followed by a leucine zipper structure. In fungi, bZIP transcription factors often regulate growth, metabolism, and environmental stress response [19–23]. For instance, eight members of the yeast activator protein (Yap) from the bZIP family are responsible for the regulation of multiple stresses, such as cadmium, oxidative stress, osmotic shock, and iron overload [24]. In *P. expansum*, a homolog of the yeast regulator Yap1 has been proven to mediate the fungus's oxidative response and to influence the expression levels of antioxidant-related genes in different isolates, according to a recent study and our unpublished research [25]. Another bZIP protein, Atf1, has been demonstrated to regulate growth, metabolism, and stress response in many fungi, including the rubber tree anthracnose fungus *Colletotrichum siamense*, the vascular wilt disease agent *Verticillium dahliae*, and *Penicillium oxalicum* [19, 26–29].

Although previous studies have investigated the function of several transcription factors in *P. expansum*, plenty of regulators remain unidentified. Besides, information on the characteristics of chromatin structure, *cis*-regulatory elements, and transcription factors in *P. expansum* is limited. In this study, we used ATAC-seq to identify open chromatin regions and *cis*-regulatory elements associated with active transcription factors during the vegetative growth and infection phases of *P. expansum*. Furthermore, we identified a bZIP transcription factor PeAtf1 and investigated its role in vegetative growth, reproduction, pathogenicity, and stress response in *P. expansum*. This study is a significant advancement in understanding the transcriptional regulatory mechanisms of *P. expansum* during growth and plant–pathogen interactions.

## Results

### Apple hosts altered chromatin accessibility in *P. expansum* during infection compared to the vegetative growth phase

**Figure 1:**
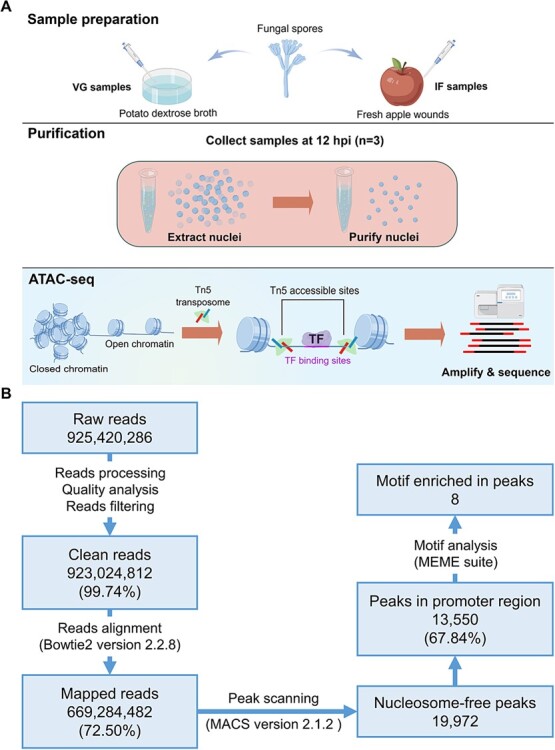
Principles and workflow of ATAC-seq for analyzing *P. expansum* during vegetative (VG) phase and infection (IF) phase. **A**, Principle and flowchart of the ATAC-seq experiment in this study. The figure was drawn using Figdraw (https://www.figdraw.com). **B**, Flowchart and results of bioinformatics analysis in ATAC-seq.

The ATAC-seq procedure can be divided into six steps: sample collection, nuclei purification, transposition, library construction, sequencing, and bioinformatics analysis. In this study, ATAC-seq was conducted to assess the chromatin accessibility of *P. expansum* during the vegetative phase (cultivated in potato dextrose broth, PDB) and the infection phase (cultivated in apple wounds), with the corresponding groups named as VG and IF, respectively (Fig. 1A). As a result, >900 million sequence reads were generated using DNA from mycelia during the VG and IF phrases (Fig. 1B). After alignment and mapping of fragments, the enriched Tn5 transposition at different genomic regions was analyzed to identify Tn5-accessible chromatin region, namely ‘peak’. A total of 19 792 high-confidence peaks were obtained after filtering, mapping, and peak scanning. Specifically, between 4180 and 5475 open chromatin regions were identified across three replicates in the VG group, while 1198–2113 peaks were identified in the IF group, respectively (Table 1). In addition to the reduced number of peaks, the IF group exhibited shorter peaks than those in the VG group. These findings suggest that the chromatin accessibility of *P. expansum* mycelia during the infection phase is lower than that during vegetative growth.

**Figure 2:**
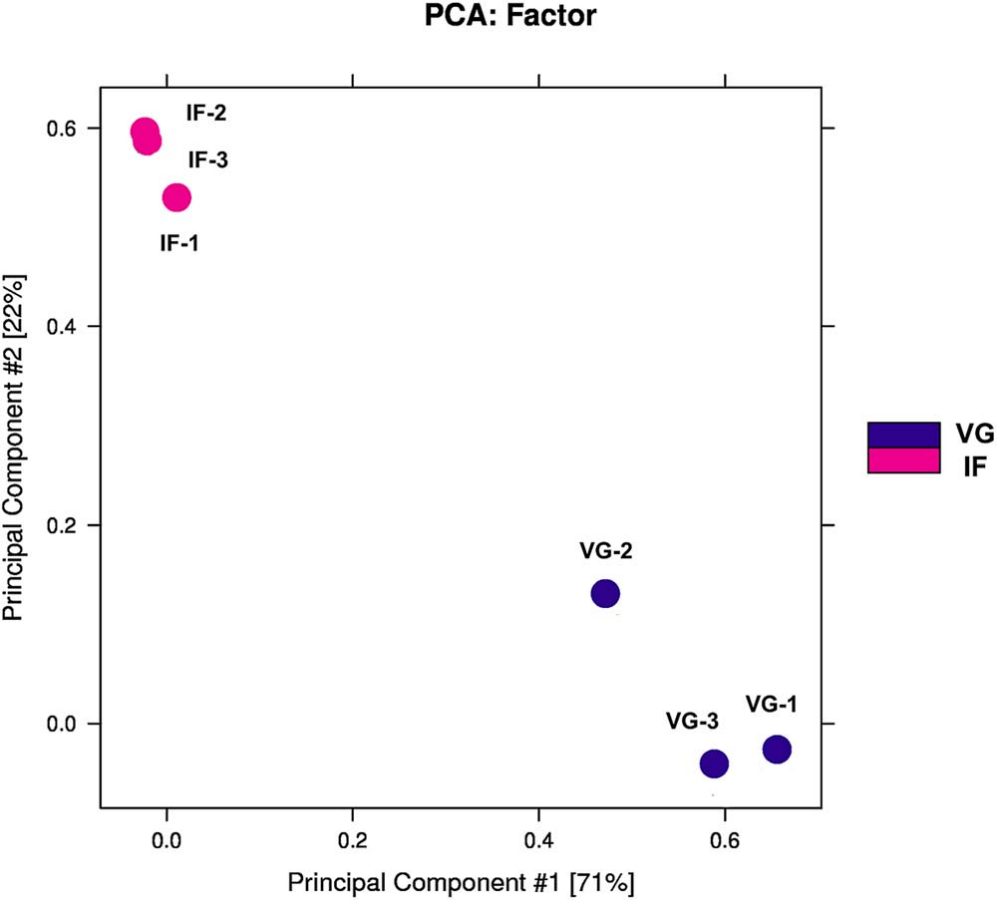
The PCA of peaks in *P. expansum* during vegetative (VG) phase and infection (IF) phase in ATAC-seq.

The principal component analysis (PCA) results for the six constructed libraries, as shown in Fig. 2, further suggest a significant difference in the chromatin accessibility landscape of *P. expansum* during vegetative growth and infection. It also indicates a strong correlation among biological replicates within each group, with samples within the same group forming clusters characterized by similar PC1 values, accounting for most of the variation (71%). These findings demonstrate that chromatin accessibility in *P. expansum* differs significantly between the infection and vegetative growth phases.

### Peaks identified by ATAC-seq were primarily located in the promoter region

**Figure 3:**
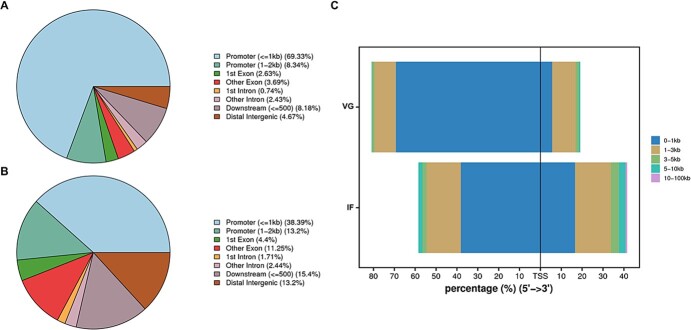
Distribution of chromatin open regions in *P. expansum* during vegetative (VG) phase and infection (IF) phase. **A**, Distribution of chromatin open regions in the VG group. **B**, Distribution of chromatin open regions in the IF group. **C**, Distribution of open chromatin regions relative to the TSS.

The distribution of peaks in the *P. expansum* genome was analyzed to elucidate the relationship between these peaks and their associated functional regions (Fig. 3). As a result, >75% of the putative peak regions in VG samples (Fig. 3A) and >50% of peaks in IF samples (Fig. 3B) were found within the promoter region, which was within 2 kb of the transcription start site (TSS). TSS is where RNA polymerase initiates transcription, and the surrounding chromatin exhibits a relatively high degree of openness. So, we then analyzed the distribution of peaks near this region. The results indicated that the majority of peaks were located within 1 kb of the TSS, particularly in the upstream promoter region (Fig. 3C). These findings suggest that the chromatin-opening region in the *P. expansum* genome across two phases is concentrated in the promoter region, underscoring its critical role in the transcriptional activation of *P. expansum*.

### Six footprint-supported motifs were enriched and matched the binding sites of known transcription factors in *Saccharomyces cerevisiae*

**Figure 4:**
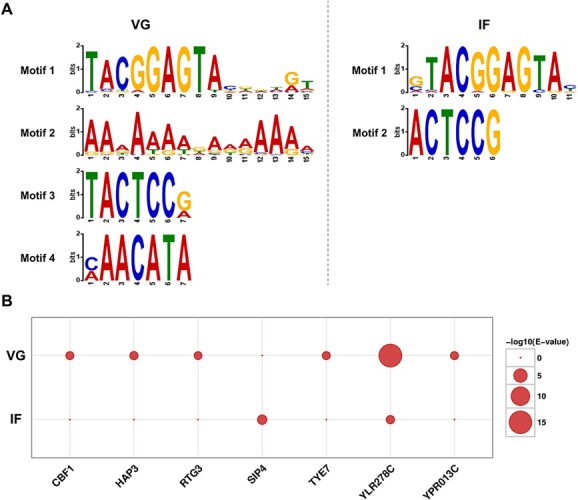
Enriched footprint-supported motifs and matched transcription factors in *P. expansum* during vegetative (VG) and infection (IF) phases. **A**, Enriched footprint-supported motifs in *P. expansum*. For the sequence logo of motifs, different colors represent different base types, and the height represents the conservatism of the base (the higher the letter, the more frequent and conservative the base occurs in this position). **B**, Matched known transcription factors by enrichment value in *P. expansum*.

Transcription factors regulate gene transcription by binding to genes in a sequence-specific manner. Based on mathematical statistical models, the conserved characteristics of these DNA sequences, or base frequency, can be expressed as motifs in a visual form. The motif analysis of peaks obtained from ATAC-seq was performed using MEME Suite (http://meme-suite.org/), where MEME detected a long motif (8–15 bp) and Dreme detected a short motif (3–8 bp). As a result, six significantly enriched motifs with E value <.5 were identified, including four from *P. expansum* at the vegetative growth stage and two from *P. expansum* at the infection stage (Fig. 4A). The different colors and heights of letters in the motifs represent different base types and conservatism.

Then, we compared motifs with the *cis*-regulatory elements of known transcription factors annotated in the JASPAR database. We found that motifs in the VG and IF group had matched six and two *cis*-regulatory elements of known transcription factors from *S. cerevisiae*, respectively (Fig. 4B). Detailed information on these motifs and corresponding transcription factors is shown in Table 2. Specifically, the motifs enriched in the VG group were similar to the DNA-binding sites of transcription factors YLR278C, HAP3, YPR013C, TYE7, CBF1, and RTG3, with motifs enriched in the IF group matching the *cis*-regulatory elements of YLR278C and SIP4. These proteins can be divided into the C_6_ zinc cluster family, CCAAT-binding factor NF-Y, C_2_H_2_ zinc finger family, and bHLH family. All transcription factors mentioned above are from the yeast species *S. cerevisiae*. This may be due to the fact that *S. cerevisiae* is the most extensively studied model organism in fungi, and the information regarding its transcription factors and *cis*-regulatory elements is relatively complete. Also, compared with animals and plants, the information about transcription factors and their motifs in fungi is still limited. Our findings may provide clues for the subsequent identification and prediction of the functions of open chromatin and transcription factors in *P. expansum*.

**Table 1:** Statistic information of peaks obtained from *P. expansum* during vegetative (VG) and infection (IF) phases in ATAC-seq

Sample	Peak number	The average length of peaks
VG-1	5475	592
VG-2	4180	514
VG-3	5086	586
IF-1	2113	304
IF-2	1198	280
IF-3	1920	316

### PeAtf1 in *P. expansum* was identified and knocked out using the homologous gene replacement strategy

**Figure 5:**
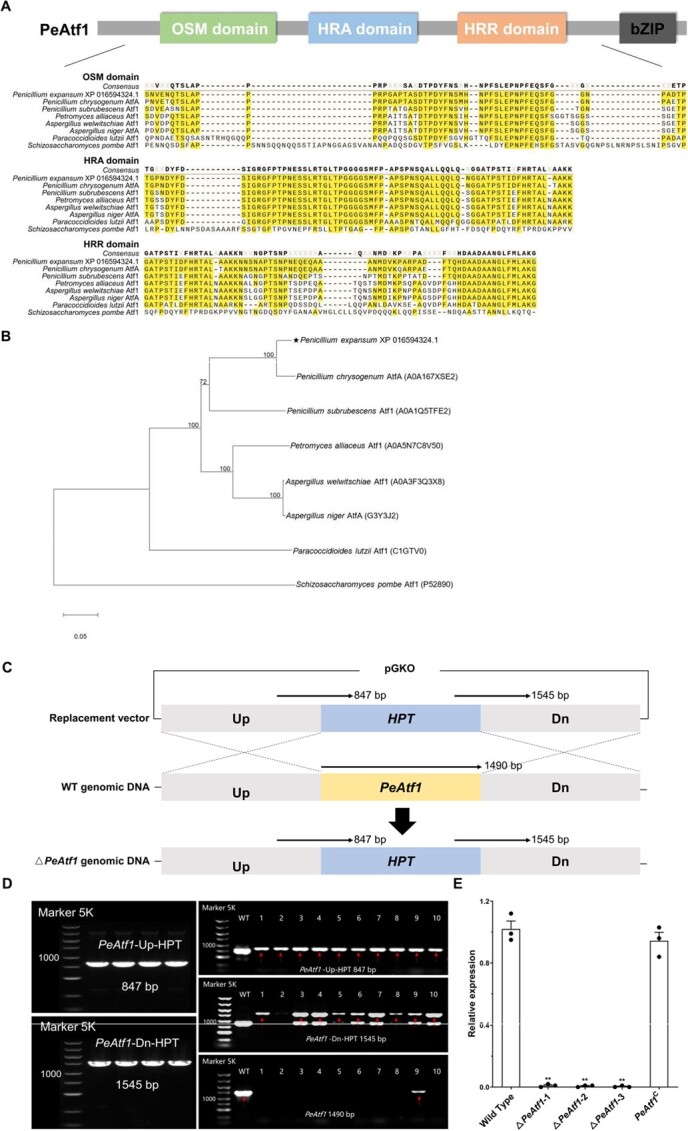
Identification, characterization, gene knockout, and complementation of *PeAtf1*. **A**, Alignment of the conserved Atf1 domains of indicated proteins. **B**, The phylogenetic analysis of indicated proteins. The horizontal bar represents the relative distance in the phylogenetic tree. The number in parentheses represents the accession number of each protein in the Uni-Prot database. **C**, Schematic illustration of the gene replacement strategy for the knockout of *PeAtf1* using the plasmid pGKO-HPT. **D**, Confirmation of the replacement vector and positive transformants by PCR. **E**, Confirmation of the gene knockout and complemented mutants by qPCR. Data represent the mean ± standard error of the mean (*n* = 3). *Significant difference at *P* < 0.05. **Significant difference at *P* < 0.01.

Transcriptional regulation of genes is influenced not only by changes in chromatin conformation but also by the behavior of transcription factors. Here, we identified PeAtf1 (gene ID: 27673634; protein ID: XP_016594324.1) as a homolog of the bZIP transcription factor Atf1/AtfA. A BLASTp search for Atf1 homologs was conducted using the Uni-Prot database. PeAtf1 displayed 94.55%, 72.28%, and 31.61% of overall identity with the transcription factor AtfA in *Penicillium chrysogenum* (A0A167XSE2), Atf1 in *Aspergillus welwitschiae* (A0A3F3Q3X8), and Atf1 in *Schizosaccharomyces pombe* (P52890), respectively. We then conducted a conserved domain analysis based on the InterPro database. Figure 5A illustrates that PeAtf1 comprises 458 amino acids, 1 bZIP domain (residues 345–410), and 3 Atf1-specific domains—OSM (residues 24–76), HRA (residues 98–167), and HRR (residues 173–238)—which share high similarity with Atf1 homologs in other fungi. Phylogenetic analysis reveals that PeAtf1 is most similar to AtfA in *P. chrysogenum* and most distant from Atf1 in the *S. pombe*, indicating that PeAtf1 may share similar biological functions with AtfA in *P. chrysogenum* (Fig. 5B).

*PeAtf1* deletion strains were then created through homologous gene replacement (Fig. 5C). A total of eight knockout mutants were generated and confirmed by polymerase chain reaction (PCR) (Fig. 5D). Three mutants from three independent experiments, namely Δ*PeAtf1*–1, Δ*PeAtf1*–2, and Δ*PeAtf1*–3, were selected for phenotypic analysis. To further validate the function of *PeAtf1*, Δ*PeAtf1*–1 was used using the same approach to generate the gene complemented mutant, which we named *PeAtf1*^C^. These mutants were validated by quantitative PCR (qPCR) to ensure the knockout and complementation of *PeAtf1* (Fig. 5E).

### PeAtf1 affected the morphology but not the pathogenicity of *P. expansum* during its invasion in apple fruits

**Figure 6:**
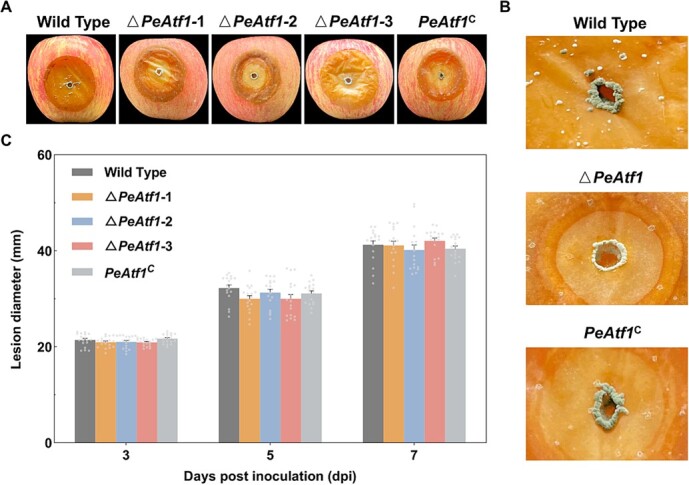
The influence of PeAtf1 on decay morphology and lesion diameter of *P. expansum*. **A**, The decay morphology of *P. expansum* wild-type and *PeAtf1* mutants at 7 days post-inoculation (dpi). **B**, The influence of PeAtf1 on the mycelial morphology of *P. expansum* in apple wounds. **C**, The influence of PeAtf1 on the lesion diameter of *P. expansum* in apple fruits at 3, 5, and 7 dpi. Data represent the mean ± standard error of the mean (*n* = 18). *Significant difference at *P* < 0.05.

As *P. expansum* can cause blue mold disease in apples, we first performed the pathogenicity experiment of the wild-type and *PeAtf1* mutants on ‘Red Fuji’ apples (Fig. 6). The absence of *PeAtf1* significantly altered the morphology of *P. expansum* mycelia at the wounded sites of the apple fruits (Fig. 6A and 6B). In contrast to the mycelia of the *P. expansum* wild type and *PeAtf1*^C^, which exhibited substantial accumulation of green conidia, Δ*PeAtf1* displayed a reduced accumulation of conidia with a lighter color (Fig. 6B). Interestingly, all *PeAtf1* mutants demonstrated similar pathogenicity to the *P. expansum* wild type throughout the observation period, as evidenced by decay lesions (Fig. 6C). These findings suggest that while PeAtf1 does not influence the pathogenicity of *P. expansum*, it does affect mycelial morphology during infection. However, whether this phenomenon is specific to the infectious process remains unclear.

### PeAtf1 positively regulates vegetative growth and reproduction in *P. expansum*

**Figure 7:**
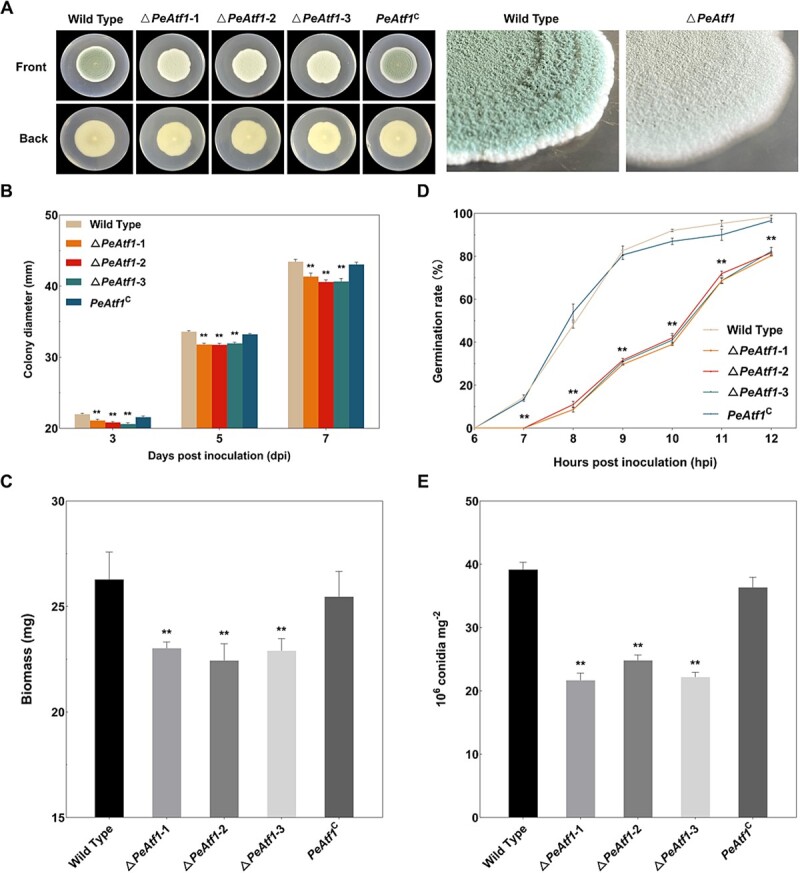
The influence of PeAtf1 on the vegetative growth and reproduction in *P. expansum*. **A**, The colony morphology of different strains on PDA at 7 dpi. **B**, The colony diameter of different strains on PDA at 3, 5, and 7 dpi (*n* = 5). **C**, The spore germination rate of different strains on PDA (*n* = 3). **D**, The biomass of different strains on PDA at 7 dpi (*n* = 5). **E**, The conidia production ability of different strains (*n* = 5). Data represent the mean ± standard error of the mean. *Significant difference at *P* < 0.05. **Significant difference at *P* < 0.01.

We compared the influence of PeAtf1 on the growth and development of *P. expansum*. Our findings demonstrated that the mycelial extension of △*PeAtf1* on potato dextrose agar (PDA) medium was significantly inhibited (Fig. 7A and 7B). Additionally, biomass measurements further support this finding, indicating that the absence of *PeAtf1* significantly reduced the biomass of *P. expansum* (Fig. 7C). Furthermore, similar to the outcome observed in Fig. 6B, the lack of *PeAtf1* noticeably changed the morphology of the *P. expansum* colony (Fig. 7A). The wild-type strain exhibited a velvety, carpet-like surface with a bluish-green color in the spore areas, whereas the Δ*PeAtf1* mutant displayed a flat and thin surface with a light gray-green color. Complementation of *PeAtf1* resulted in the restoration of the observed growth defects.

To investigate whether the growth defects of Δ*PeAtf1* are related to spore development, we compared the spore germination rates and reproductive capabilities of the indicated strains. Our results revealed that Δ*PeAtf1* exhibited a significantly delayed spore germination process (Fig. 7D) and approximately half the spore production capacity (Fig. 7E) compared with the wild-type strain. Given that the green coloration of the colony results from spore accumulation, the reduced reproductive ability of Δ*PeAtf1* may explain the previously observed alterations in colony morphology shown in Fig. 6B and 7A. These findings suggest that PeAtf1 positively regulates the vegetative growth and reproduction of *P. expansum* by influencing the processes of spore germination and production.

### PeAtf1 positively regulates tolerance to osmotic stress while negatively regulating tolerance to membrane, cell wall, and oxidative stresses in *P. expansum*

**Figure 8:**
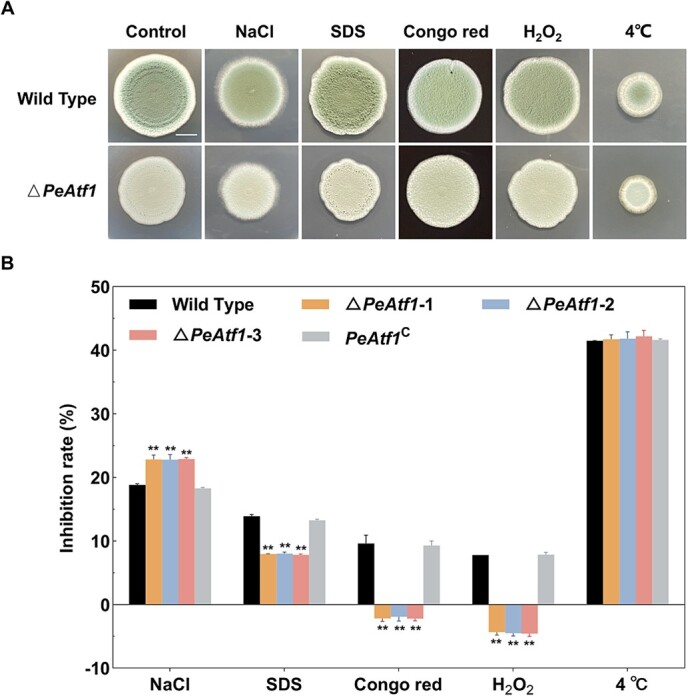
The effect of PeAtf1 on the tolerance of *P. expansum* to different stress conditions. **A**, The morphology of different strains under various stresses at 7 dpi. Scale bar = 10 mm. **B**, The growth inhibition rate of different strains under various stresses. NaCl (1 M), SDS (0.1 g/l), Congo red (3 g/l), H2O2 (1 mM), and the growth temperature of 4°C were used to produce osmotic, membrane, cell wall, oxidative, and cold stresses, respectively. The growth inhibition rate = [(diameter of untreated strain – diameter of treated strain) / (diameter of untreated strain × 100%)]. Data represent the mean ± standard error of the mean (*n* = 3). *Significant difference at *P* < 0.05. **Significant difference at *P* < 0.01.

Considering the significance of stress response in plant–pathogen interactions, we investigated whether PeAtf1 impacted stress response in *P. expansum*. Five stressors, including osmotic stress (NaCl), membrane stress (SDS), cell wall stress (Congo red), oxidative stress (H_2_O_2_), and cold stress (growth temperature of 4°C), were applied to each strain. The colony diameter of each strain grown on the PDA medium served as the control (Fig. 8A). Regarding stress tolerance, deleting *PeAtf1* significantly increased fungal sensitivity to high osmotic stress (Fig. 8B). In contrast, the sensitivity of *P. expansum* to cell membrane, cell wall, and oxidative stresses was markedly reduced with no significant changes observed in response to cold stress, when compared to the wild type and *PeAtf1*^C^. Notably, the wild type exhibited an inhibition rate of ~10% under oxidative stress, whereas Δ*PeAtf1* demonstrated an inhibition rate of around −5%, highlighting the negative role of PeAtf1 in the fungus's tolerance to reactive oxygen species (ROS) in *P. expansum*.

**Figure 9:**
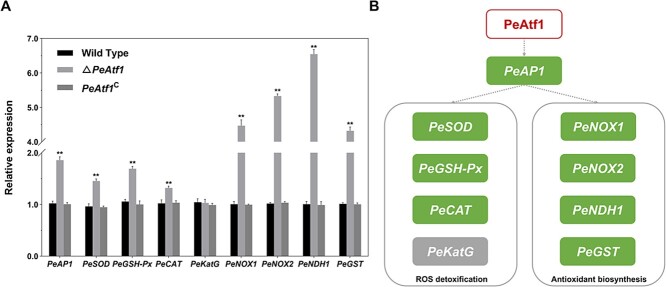
The effect of PeAtf1 on the expression of genes participating in the fungus's response to oxidative stress. **A**, The relative expression of associated genes in different strains. Data represent the mean ± standard error of the mean (*n* = 3). *Significant difference at *P* < 0.05. **Significant difference at *P* < 0.01. **B**, The schematic diagram of the role of PeAtf1 in regulating genes regarding ROS detoxification and antioxidant biosynthesis.

To clarify whether PeAtf1 regulates oxidative stress-related genes, we further measured the expression levels of these genes in different *P. expansum* strains. The results presented in Fig. 9 indicate that *PeAtf1* deletion resulted in approximately a 2-fold increase in the expression of the core oxidative stress response regulator *PeAP1*, which positively influenced the expression of genes associated with oxidative stress response. Furthermore, downstream genes of PeAP1 involved in ROS detoxification (*PeSOD*, *PeGSH-Px*, and *PeCAT*) showed a significant increase after *PeAtf1* deletion. Notably, four genes related to antioxidant biosynthesis were all dramatically up-regulated by at least 4-fold in △*PeAtf1*. These results further prove that PeAtf1 negatively regulate oxidative stress response in *P. expansum* by governing the expression of multiple genes regarding oxidative stress response.

## Discussion

The postharvest fungus *P. expansum* is the causal agent of blue mold and has caused severe rot and mycotoxin contamination in fruits and fruit-based products globally [30]. Understanding the molecular mechanisms of *P. expansum* during growth and infection is the basis for developing management strategies to control this pathogen. In this study, we used ATAC-seq to investigate the landscape of chromatin accessibility in *P. expansum* during vegetative growth and the plant–pathogen interaction phase. Then, we identified a transcription factor, PeAtf1, and explored its regulatory role in the growth, stress response and pathogenicity of *P. expansum*.

### Chromatin accessibility in *P. expansum* during vegetative growth and infection phases

Our previous study investigated the transcriptome of *P. expansum* and identified many genes with differential expression in *P. expansum* during the vegetative growth and infection phase [16]. In this study, we analyzed the characteristics of *P. expansum* during these two phases at the level of chromatin conformation. When applying ATAC-seq to microorganisms, DNA contamination from plants, bacteria, and organelles is a common problem that can lead to low reads mapping [4, 31]. Our data achieved a mapping rate of 72.05%, which ensures a relatively high quality of sequencing (Fig. 1).

**Table 2:** Information of matched known transcription factors in the JASPER database by motif analysis

**Motif ID**	**Motif**	**Transcription factor**	**Family**
MA0430.1	YCGGAGTT	YLR278C	C_6_ zinc cluster family
MA0314.1	TCTSATTGGYYVRRA	HAP3	CCAAT-binding factor NF-Y
MA0434.1	YGTARATCM	YPR013C	C_2_H_2_ zinc finger family
MA0409.1	CACGTGA	TYE7	bHLH family
MA0281.1	GCACGTGA	CBF1	bHLH family
MA0376.1	RDVKKAGCACGTGCYYDNWH	RTG3	bHLH family
MA0380.1	YTCCGGA	SIP4	C_6_ zinc cluster family

Furthermore, our analysis revealed that the chromatin accessibility of *P. expansum* changed significantly when transiting from the vegetative growth stage to the infection stage (Fig. 2). This finding aligns with our previous study at the transcriptomic level, which identified significant differences in gene expression in *P. expansum* during these two phases [16]. We actually also conducted an integrative analysis of ATAC-seq and RNA-seq to investigate the relationship between peak number and gene expression; however, we found their relevance to be relatively low (with a Pearson correlation coefficient ranging from 0.2 to 0.3), and thus these results were not presented. A similar phenomenon was observed in *P. sojae*, where the Pearson correlation coefficient was slightly higher at 0.33 [4]. These studies suggest that ATAC-seq may currently not be suitable for associating gene expression with the number of peaks in plant pathogens compared to animals and plants. Moreover, open chromatin regions were concentrated in the promoter regions near TSS in this study (Fig. 3). This is reasonable as the promoter region surrounding the TSS is where gene transcription initiates, for it contains multiple *cis*-elements that can be recognized by transcription factors [5]. Similar outcomes have been reported in *Aspergillus* species and the rice pathogen *Ustilaginoidea virens* [10, 31].

### The characteristics of conserved motifs in *P. expansum* during vegetative growth and infection phases

Based on the distribution characteristics of the fragment sizes obtained by ATAC-seq, major sequence fragments can be classified into signals from regions with or without nucleosomes. Since transcription factors are more likely to bind to nucleosome-free regions, we focused on these regions to discover transcription factor-binding motifs. Our study revealed a total of six enriched motifs, comprising four from the VG group and two from the IF group (Fig. 4). The reduced ative chromatin accessibility observed in the IF group, relative to the VG group, may account for the smaller number of identified motifs [32]. Several motifs have matched in the JASPER database to *cis*-regulatory elements of known transcription factors in *S. cerevisiae* (Table 2). The motif feature of the C_6_ transcription factor YLR278C is enriched in both VG and IF groups. YLR278C is not necessary for the growth of *S. cerevisiae* but can positively regulate fungal tolerance to caffeine [33, 34]. It is also linked to ribosomal DNA, suggesting a potential role in protein translation [35]. The motif of another C_6_ transcription factor, SIP4, is only enriched in the IF group. It can be induced by environmental glucose and activates the carbon source-responsive element [36, 37]. Furthermore, TYE7 is associated with the glycolytic process and sulfur metabolism, while CBF1 regulates cell respiration and chromatin conformation [38–42]. HAP3, YPR013C, and RTG3 in *S. cerevisiae* are associated with the regulation of respiratory gene expression, carbon source response, and mitochondrial stress response [43–45].

However, due to limited information on identified *cis*-regulatory elements in fungi, all transcription factors matched in our study are from *S. cerevisiae*. Considering the relatively far evolutionary distance between *S. cerevisiae* and *P. expansum*, finding corresponding homologs of these transcription factors and validating their role in *P. expansum* would be difficult. Our results contribute to the identification of *cis*-regulatory elements in *P. expansum*. In the future, further studies of fungal transcription factors and their motifs are needed to provide information for experimental validation of protein functions and protein–gene interactions.

### The role of PeAtf1 in the growth and development of *P. expansum*

The life cycle of *P. expansum* begins with the germination of asexual spores (conidia), followed by extension and subsequent spore production [16]. In this study, the deletion of *PeAtf1* severely retarded both the growth and formation of conidia, which further impaired the mycelial growth and development of *P. expansum* (Fig. 7). This finding is consistent with previous observations regarding colony diameter and spore germination in *Atf1* deletion mutants of *V. dahliae*, *C. siamense*, and *Trichoderma guizhouense* [19, 26, 46]. Additionally, a similar regulatory role of Atf1 homologs in fungal reproduction has been demonstrated in *Botrytis cinerea*, *V. dahliae*, *C. siamense*, and *Aspergillus nidulans* [19, 26, 47, 48].

Given the crucial role of conidia in the transmission of *P. expansum* during infection, it is surprising that the knockout of *PeAtf1* did not alter the virulence of *P. expansum* on fruit hosts [11]. The orthologs of Atf1 in several fungi, such as *Fusarium graminearum*, *V. dahliae*, and *C. siamense*, have been proven to positively regulate fungal pathogenesis [19, 26, 47]. Conversely, knocking out *BcAtf1* resulted in increased colonization efficiency of *B. cinerea* on different host plants [47]. Our results indicate that the pathogenicity of *P. expansum* on hosts results from multiple factors.

### The role of PeAtf1 in the response of *P. expansum* to multiple environmental stresses

Plant pathogens are exposed to various stimuli during their interactions with plant hosts, such as oxidative burst, hyperosmolarity, and phytoalexins. The ability of fungi to adapt to these stresses is crucial for their survival and successful invasion. Among these stresses, the oxidative burst generated by plant hosts is particularly challenging for the infection of pathogens [49]. Our results indicated that the absence of *PeAtf1* significantly enhanced the tolerance of *P. expansum* to oxidative stress (Fig. 8). Consistently, Atf1 homologs exhibit a negative regulatory effect on the tolerance of *C. siamense* and *F. graminearum* to H_2_O_2_ stress [26, 50]. In *T. guizhouense* and *Aspergillus flavus*, Atf1 is essential for fungal resistance to oxidative stress, although it displays an opposing effect [21, 46]. In addition, a recent study has proven that PeAP1 serves as the core regulator of the oxidative stress response in *P. expansum* and controls the expression of antioxidant-related downstream genes [25]. We noticed that *PeAtf1* deletion effectively induced the expression of *PeAP1*, along with its downstream genes participating in ROS detoxification and antioxidant biosynthesis, such as *PeSOD*, *PeGSH-Px*, *PeCAT*, and *PeGST* (Fig. 9). Notably, PeGST has been proven to positively influence the growth and pathogenicity of *P. expansum* [25]. Therefore, it is possible that the increased tolerance of Δ*PeAtf1* to oxidative stress, coupled with the enhanced expression of virulence-related genes, contributes to the pathogenicity observed in *PeAtf1* deletion mutants.

Similar to the results of oxidative stress test, the absence of *PeAtf1* enhanced the tolerance of *P. expansum* to both cell wall and membrane stresses (Fig. 8). Consistent results were observed in *C. siamense* [26]. Likewise, in *A. nidulans*, Δ*AtfA* displayed increased tolerance to cell wall stress [48]. But in *B. cinerea*, Atf1 displayed an opposing role in fungal response to cell wall stress [47]. In addition, Atf1 has been studied in many fungi regarding its role in regulating osmotic stress response. For instance, in *A. nidulans* and *Penicillium digitatum*, Atf1 did not influence the fungal response to a high osmotic environment [23, 48]. In *C. siamense*, knocking out *CsAtf1* significantly reduced fungal sensitivity to osmotic stress [26]. Conversely, *F. graminearum*, after deleting *Atf1*, shows more sensitivity to high osmotic pressure, which is supported by our results [50]. These findings collectively indicate that Atf1 plays a conserved role in the stress response, although the specific regulatory mechanisms may differ among various fungal species. 

## Conclusion

In this study, we identified the landscape of chromatin accessibility and motif characteristics in *P. expansum* during the vegetative growth and infection phases using ATAC-seq. We demonstrated a significant difference in chromatin accessibility between these two phases and highlighted the role of the promoter region in transcription regulation in *P. expansum*. The exploration of footprint-supported motifs and matched transcription factors shown in our results will aid in the discovery of new regulatory factors and their potential sites of action. More information on the binding sites of fungal regulators is needed when applying ATAC-seq to screen candidate transcription factors in fungi in the future. Additionally, we identified a bZIP transcription factor PeAtf1 and explored its role in *P. expansum*. Despite the observed defects in growth and development in *PeAtf1* deletion mutants, the enhanced tolerance to oxidative stress and expression of genes participating in the fungus's response to oxidative stress in deletion mutants may contribute to fungal pathogenicity, leading to similar virulence between ∆*PeAtf1* and the wild type. Furthermore, PeAtf1 exerted a negative regulatory effect on the fungal response to cell wall and membrane stress while demonstrating a positive effect on the response to high osmolarity. Our results suggest that PeAtf1 serves not only as a positive regulator of growth and development, but also as a key controller in *P. expansum* responding to environmental stresses.

## Materials and methods

### Preparation of fungal spore suspension and apple fruits

As previously described, spores from *P. expansum* grown on a PDA plate were collected using sterile distilled water [16]. The spore suspension was then filtered, and the hemocytometer method was used to adjust it to the final concentration. The fruits of the apple cultivar (*Malus domestica* Borkh. cv. Red Fuji) were purchased, selected, hand-washed, immersed, rewashed, and finally air-dried at room temperature. Three uniform wounds (4 × 4 mm) were created by a sterile stainless-steel cylinder at the equator of each apple fruit.

### Sample preparation, library construction, and sequencing of ATAC-seq

Samples were collected following the procedure described in our previous study [16]. After inoculating each wound with 30 μl of the spore suspension (1 × 10^8^ spores/ml), apple fruits were kept in baskets. The 1% spore suspension (1 × 10^8^ spores/ml) of *P. expansum* was cultivated in PDB. Mycelia samples were harvested at 12 h post-inoculation (hpi), with mycelia grown in PDB as the VG group and mycelia grown in apple wounds as the IF group, respectively. At least 36 samples from 36 apples from three independent experiments were collected and stored at −80°C. The nuclei suspension, transposition reaction, PCR amplification, purification, library construction, and sequencing were conducted by Gene Denovo Biotechnology Co. (Guangzhou, China).

### Bioinformatics analysis of ATAC-seq

The bioinformatics analysis, including quality analysis, alignment, peak analysis, and motif analysis in ATAC-seq, was performed following the previously described method [51]. High-quality reads were aligned to the *P. expansum* reference genome (https://www.ncbi.nlm.nih.gov/assembly/GCF_000769745.1/) with mitochondrial and chloroplast sequences excluded using Bowtie2 (version 2.2.8). Peak calling was conducted by MACS (version 2.1.2). MEME suit (http://meme-suite.org/) was used to detect the motifs. Characteristics of motifs were compared to DNA-binding sites of known transcription factors in the JASPER database (https://jaspar.elixir.no/).

### Sequence and conserved domain analysis of PeAtf1

Nucleotide and protein sequences of PeAtf1 (gene ID: 27673634; protein accession number: XP_016594324.1) were both downloaded from the NCBI website (https://www.ncbi.nlm.nih.gov/). Atf1 homologs with accession numbers were downloaded from the Uni-Prot database (https://www.uniprot.org/). The conserved domains of the proteins were analyzed using InterPro (https://www.ebi.ac.uk/interpro/). SnapGene version 5.2 software was utilized to conduct similarity and multiple sequence alignments for full-length protein sequences. The Clustal W in MEGA version 11.0 software was applied to align protein sequences for constructing the Neighbor-Joining phylogenetic tree. Default settings and 1000 bootstrap replications were used in the analysis.

### Construction of gene knockout and complemented mutants

The construction of gene knockout and complemented mutants was carried out using *Agrobacterium*-mediated transformation and a homologous recombination strategy, following previously described methods [52]. Briefly, the upstream and downstream homologous arms were separately amplified from *P. expansum* genomic DNA, purified, and inserted into the pGKO-HPT vector to create the recombinant plasmid. This plasmid was validated through PCR and gene sequencing before being transferred into *Agrobacterium tumefaciens* EHA 105 for homologous recombination in co-culture with *P. expansum*. Transformants were selected using Hygromycin B (250 μg/ml) through three independent transformations. Three independently positive transformants were named △*PeAtf1*–1, △*PeAtf1*–2, and △*PeAtf1*–3 for subsequent phenotypic analysis. When generating complemented strains, the full-length sequence containing homologous arms and *PeAtf1* was cloned and inserted into pGKO-HPT. The deletion mutant ∆*PeAtf1*–1 was used in co-culture to generate complemented strains.

All transformants were individually isolated using single-spore isolation and further validated through PCR and qPCR.

### Phenotypic analysis

(1) Pathogenicity

Selected apple fruits were washed and wounded (4 × 4 mm) at the equator. Each wound was inoculated with a 10-μl spore suspension (1 × 10^6^ spores/ml). Apple fruits were then placed in baskets and kept at a temperature of 25°C. The diameter of the lesions on fruits was measured by recording two perpendicular diameters at 3, 5, and 7 dpi. Eighteen fruits were used for each strain.

(2) Colony morphology and colony diameter

Two microliters of a spore suspension (1 × 10^6^ spores/ml) was spotted onto PDA medium and then incubated at 25°C in the dark. The colony diameter was measured by assessing two perpendicular diameters of each colony at 3, 5, and 7 dpi. Five plates were used per strain.

(3) Conidial germination rate

Fifty microliters of a spore suspension (5 × 10^7^ spores/ml) was evenly spread on PDA plates. The percentage of germinated spores was determined using a NE910 microscope from Nexcope (Ningbo, China) at 6–12 hpi in the dark at 25°C. A total of 100 conidia were counted per plate, with three plates analyzed for each strain.

(4) Biomass

Two microliters of a spore suspension (1 × 10^6^ spores/ml) was spotted onto a cellophane sheet (80 × 80 mm), which was placed on a PDA medium and then incubated in the dark at 25°C for 7 days. The mycelia were collected, dried at 65°C for at least 48 h, and weighed. Five plates were used per strain.

(5) Sporulation

Two microliters of a spore suspension (1 × 10^6^ spores/ml) was spotted onto a cellophane sheet (80 × 80 mm), which was placed on a PDA medium and then incubated in the dark at 25°C for 7 days. Conidia from each colony were harvested by adding 0.05% Tween 20 and filtering through lens wiping paper. The volume of spore suspensions was measured, and the concentration was determined using a hemocytometer. The mycelia were collected, dried at 65°C for at least 48 h, and weighed. Five plates were used per strain.

(6) Stress tolerance

For the control, strains were spotted on PDA plates for 7 days. For stress treatments, strains were treated with PDA plates containing NaCl (1 M), sodium dodecyl sulfate (SDS, 0.1 g/l), Congo red (3 g/l), and H_2_O_2_ (1 mM) to induce osmotic, membrane, cell wall, and oxidative stress, respectively. Aliquots of 2-μl spore suspensions (1 × 10^6^ spores/ml) were then spotted onto different plates and incubated in the dark at 25°C. At 3 dpi, strains grown on PDA were incubated at 4°C to induce cold stress. At 7 dpi, the colony diameter was measured by assessing two perpendicular diameters of each colony. Inhibition rate = [(diameter of untreated strain − diameter of treated strain) / (diameter of untreated strain × 100%)]. Three plates were used per strain for each treatment.

### Reverse transcription and qPCR analysis

Two microliters of a spore suspension (1 × 10^6^ spores/ml) was spotted onto a cellophane sheet (10 × 10 mm), which was placed on a PDA medium and then incubated in the dark at 25°C for 3 days. The mycelia were collected, frozen in liquid nitrogen for 5 min, and stored at −80°C. Three cellophane sheets were used per strain. RNA extraction and reverse transcription were conducted as described previously [53]. The qPCR analysis was conducted on a LightCycler® 96 System (Roche, Switzerland) using ChamQ SYBR qPCR Master Mix (Vazyme, China) with three biological replicates and three technical duplicates. The 2^-△△Ct^ method was utilized to determine the relative expression levels of the genes after normalizing them to the *β-tubulin*. The primers used in experiments are listed in Table S1 and S2 in Supplementary information.

### Statistical analysis

The one-way analysis of variance (ANOVA) in SPSS software version 17.0 (SPSS Inc., Chicago, Illinois, USA) was performed to assess mean differences between groups. The difference at *P* < 0.05 was considered significant by the least significant difference. Duncan's multiple range test (*P* < 0.05) was used for mean comparison.

## Supplementary Material

Web_Material_uhae264

## Data Availability

ATAC-seq data are available in the National Genomics Data Center (NGDC) under BioProject accession PRJCA029681. Other data are included in this article and the supplementary data.
