# Supplementary material for: Chromatin accessibility profile and the role of PeAtf1 transcription factor in the postharvest pathogen Penicillium expansum
Source: Hortic Res. 2024 Sep 20;12(1):uhae264. doi: 10.1093/hr/uhae264 (PMC11718402; doi:10.1093/hr/uhae264)
Supplement: Web_Material_uhae264 [file web_material_uhae264.zip › Supplementary file for review.docx]

**Supplementary file for review**

An integrative analysis of gene expression derived from ATAC-seq and RNA-seq data was conducted. While a positive relationship between gene expression from ATAC-seq and RNA-seq was observed, the correlation was relatively low, with an R-value of 0.29 (Fig. 1). The analysis of the relationship between the transcription levels of genes and the number of peaks further supports this finding (Fig. 2). Overall, genes associated with open chromatin exhibited higher average transcription levels than those associated with closed chromatin, indicating that chromatin-accessible regions play a role in the transcriptional activation of their target genes.


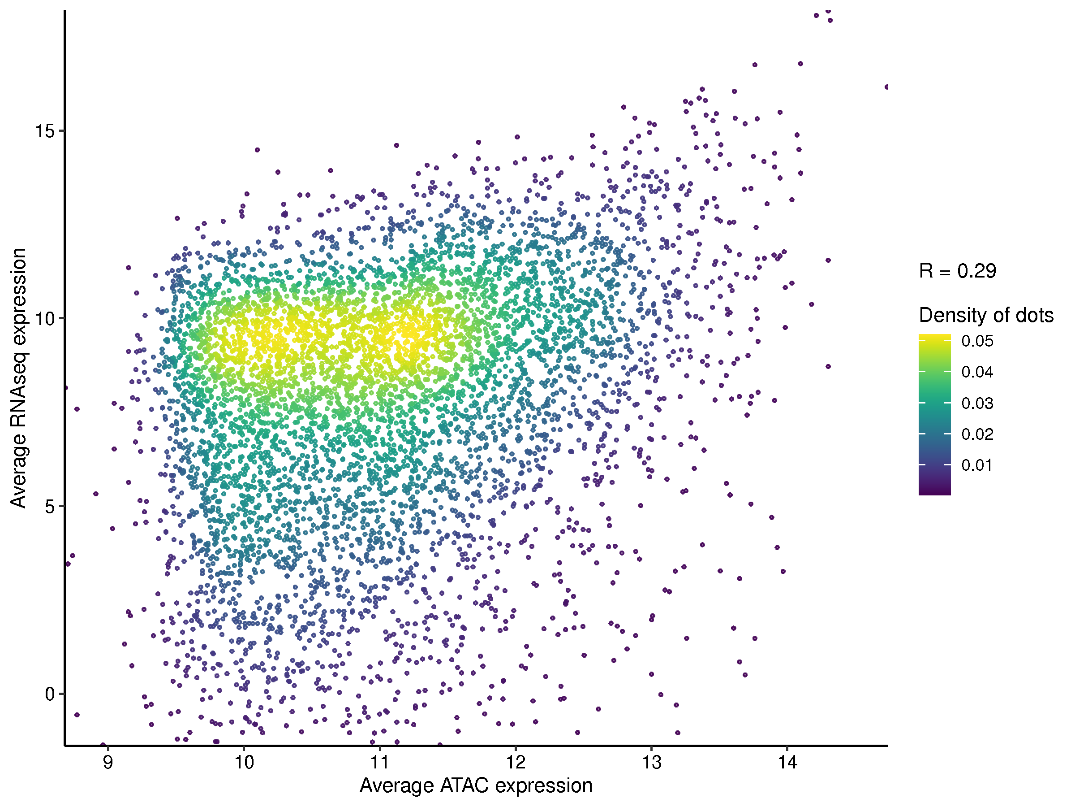


**Fig 1.** Correlation analysis between gene expression level from RNA-seq and ATAC-seq. The Pearson correlation coefficient (R) is shown.


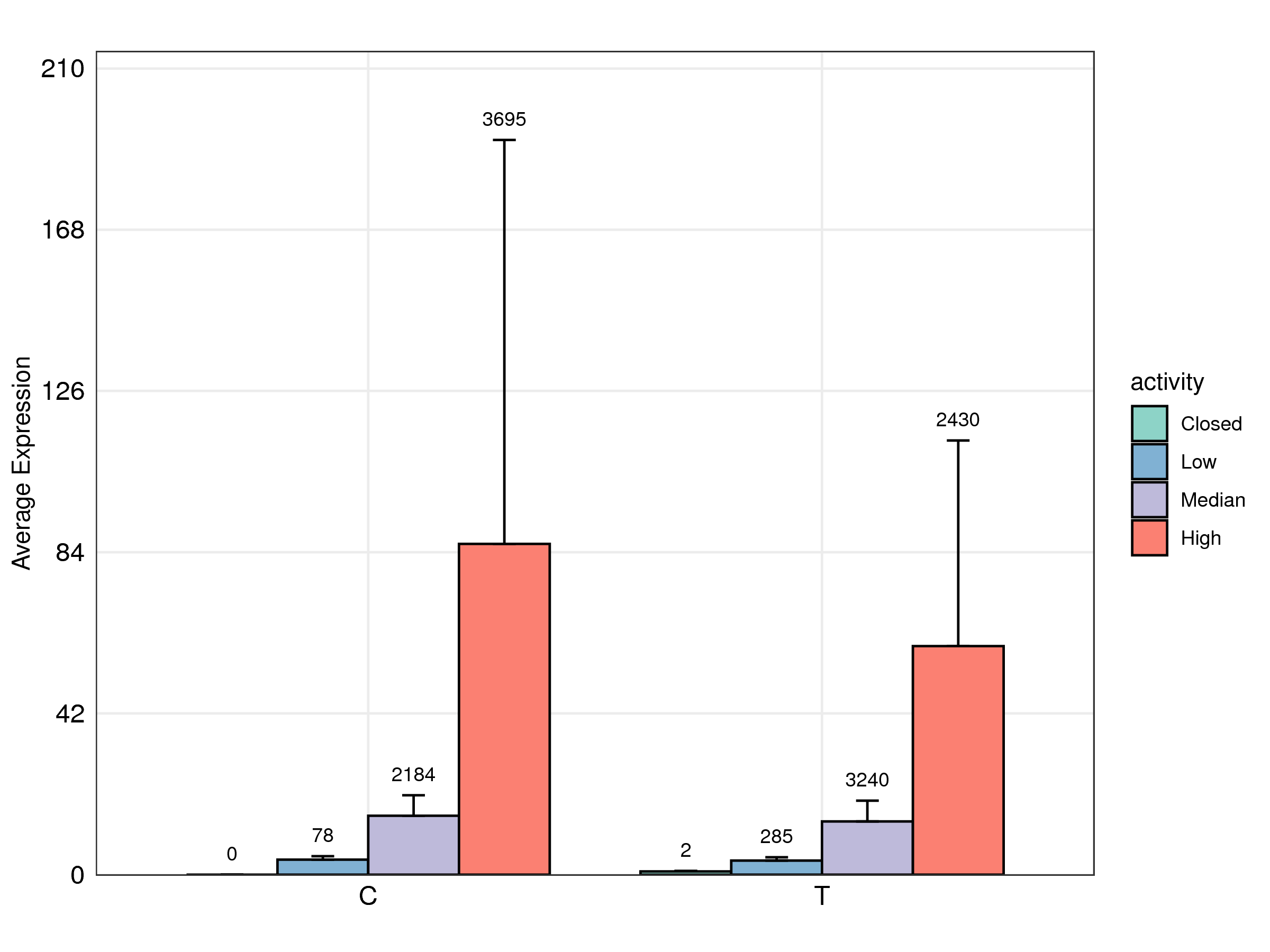


**Fig 2.** Comparison of gene transcription levels according to peak number in the VG (left) and IF (right) group. Data represent the mean ± standard deviation (SD). Numbers accompanying SD is the gene number. *Significant difference at *P* < 0.05. **Significant difference at P < 0.01. Closed: RPKM <= 1. Low: 1 < RPKM <= 5. Med: 5 < RPKM <= 25. High: RPKM > 25.

Our results demonstrated that the deletion of *PeAtf1* positively influenced the expression of *PeAP1*. We constructed gene deletion and complementation mutants of *PeAP1* (Fig. 3). According to our unpublished data, in contrast to the role of PeAtf1, PeAP1 acts as a positive regulator of the oxidative response and has a more significant impact on the tolerance of *P. expansum* to reactive oxygen species (ROS). Additionally, the downstream genes of PeAP1 were all down-regulated after the deletion of *PeAP1* (Fig. 4).


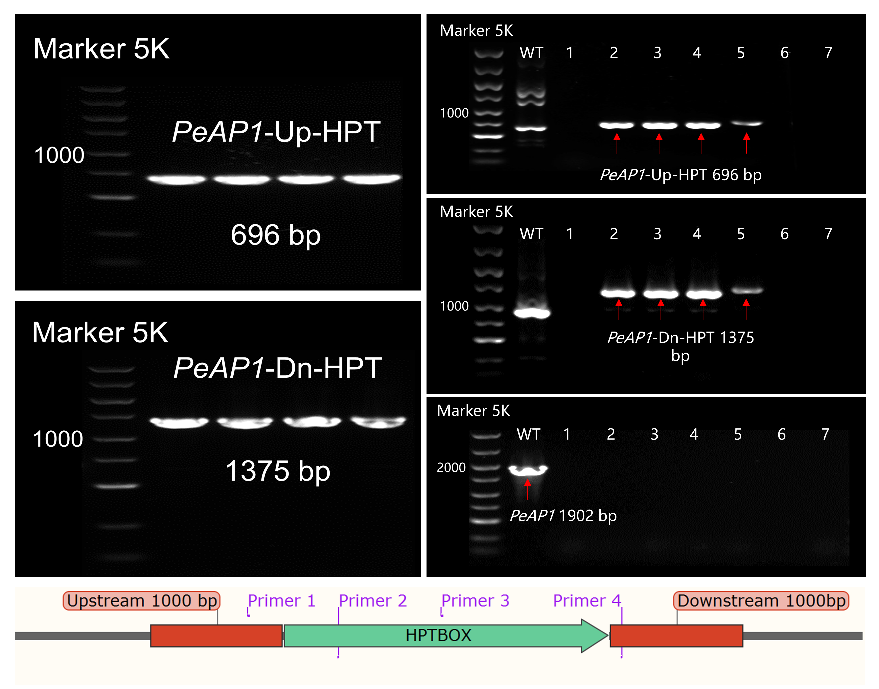


**Fig 3.** PCR validation of recombinant plasmids and transformants during *PeAP1* deletion. Primer 1-4: the validation primers for recombined plasmid.


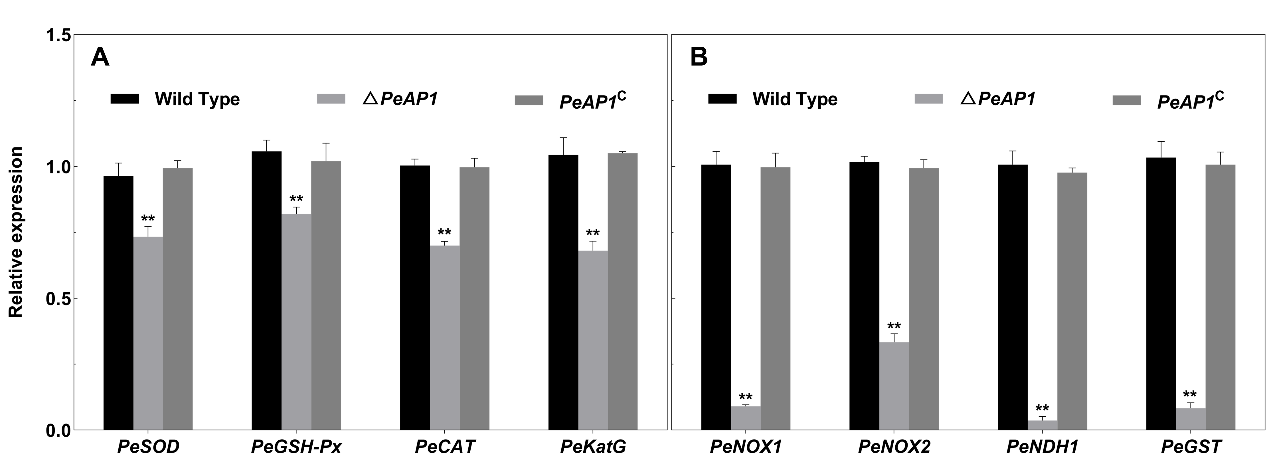


**Fig 4.** The effect of PeAP1 on the expression of oxidative stress-related genes. Data represent the mean ± standard error of the mean (n = 3). *Significant difference at *P* < 0.05. **Significant difference at *P* < 0.01
