# Supplementary material for: Chromatin accessibility profile and the role of PeAtf1 transcription factor in the postharvest pathogen Penicillium expansum
Source: Hortic Res. 2024 Sep 20;12(1):uhae264. doi: 10.1093/hr/uhae264 (PMC11718402; doi:10.1093/hr/uhae264)
Supplement: Web_Material_uhae264 [file web_material_uhae264.zip › Supplementary Table S1 Sequence of primer pairs used for the gene knockout and complementation of PeAtf1 in P. expansum.docx]

**Table S1.** Sequence of primer pairs used for the gene knockout and complementation of *PeAtf1* in *P. expansum*.

| **Primers** | **Primer sequences (5'-3')** | **Purpose** |
| --- | --- | --- |
| PeAtf1-U-F | TTGGAACTCCCCCAGGGTATC | Cloning of *PeAtf1* upstream homologous arm (1000 bp) |
| PeAtf1-U-R | TGTGTGTGGAAGATGCATGTGT |  |
| PeAtf1-D-F | GAAAAGCTGTTTTGTTTCTCATCTC | Cloning of *PeAtf1* downstream homologous arm (1000 bp) |
| PeAtf1-D-R | AACCCGATACTCTACCCCAGC |  |
| PeAtf1-LU-F | ACTGGCGTCATTGATTTCCT | Confirmation of the linkage of upstream homologous arm and HPT (847 bp) |
| PeAtf1-LU-R | CCGCTCGTCTGGCTAAGAT |  |
| PeAtf1-LD-F | CTCGCCGATAGTGGAAACC | Confirmation of the linkage of downstream homologous arm and HPT (1545 bp) |
| PeAtf1-LD-R | ATGTGCCCTCAAGTATTAGACAA |  |
| PeAtf1-F | ATGAAGCCCCCGACTCGATC | Detecting of *PeAtf1* (1490 bp) |
| PeAtf1-R | TCATCGACGCATGCCCTGTG |  |
| PeAtf1-FL-F | GAGCTCGCTGAGGGTTTAATTAATTGGAACTCCCCCAGGGTAT | Cloning full length for complementation (3490 bp) |
| PeAtf1-FL-R | AAGCTTGCTGAGGTCTTAATTAAAACCCGATACTCTACCCCAGCA |  |
| β-Tublin-q-F | CTCCAGCTCGAGCGTATGAAC | The internal gene for qPCR analysis |
| β-Tublin-q-R | GGCTCCAAATCGACGAGAAC |  |
| PeAtf1-q-F | GGCAAGAAGAACACCGCTAAGG | Confirmation of the deletion and complementation of *PeAtf1* by qPCR analysis |
| PeAtf1-q-R | CATCATCCATATCCGAAGGCATCTC |  |
