# Supplementary material for: Chromatin accessibility profile and the role of PeAtf1 transcription factor in the postharvest pathogen Penicillium expansum
Source: Hortic Res. 2024 Sep 20;12(1):uhae264. doi: 10.1093/hr/uhae264 (PMC11718402; doi:10.1093/hr/uhae264)
Supplement: Web_Material_uhae264 [file web_material_uhae264.zip › Supplementary Table S2 Sequence of primer pairs used for the qPCR of oxidative-stress response genes.docx]

**Table S2.** Sequence of primer pairs used for the qPCR of oxidative-stress response genes.

| **Gene** | **Primer name** | **Primer sequences (5’→3’)** |
| --- | --- | --- |
| *β-tubulin* | F | CTCCAGCTCGAGCGTATGAAC |
|  | R | GGCTCCAAATCGACGAGAAC |
| *PeAP1* | F | ATGGGTGATTACGAACGATTCC |
|  | R | CTACTTGGCTCGTCCCATAATG |
| *PeSOD* | F | CCCCGAACATGACCCTCAT |
|  | R | TCCCGCTTGACGGATAGTTG |
| *PeGSH-Px* | F | GGCTCGGACGATGATATCCA |
|  | R | TTCACATCCAGTTTTCCGAGAA |
| *PeCAT* | F | ACGCTCCACACCCTTCTTTG |
|  | R | TGCACACCGAATCCATCAAC |
| *PeKatG* | F | GTCAACAACCCAGCCCAGTT |
|  | R | CCATTCTGGGCACTGTTGAA |
| *PeNOX1* | F | AATCCGAAGCCGCAGTTTG |
|  | R | CAGTGGTCGGCTCAACCAA |
| *PeNOX2* | F | GGAGCTGATCCACGCCTACA |
|  | R | CGGCATCGATCATGACATTG |
| *PeNDH* | F | CCCCACTCGAGACATTGCTT |
|  | R | TTTCATTGGCAATCCGGAAT |
| *PeGST* | F | ACAGCGACCGGGTACTGAGA |
|  | R | CAGCCGCCTTATCCTTGATG |

.
